# Stochastic modeling of biochemical systems with multistep reactions using state-dependent time delay

**DOI:** 10.1038/srep31909

**Published:** 2016-08-24

**Authors:** Qianqian Wu, Tianhai Tian

**Affiliations:** 1 School of Mathematical Sciences, Monash University, Melbourne, VIC 3800, Australia; 2 School of Mathematics Hefei University of Technology, Hefei, Anhui 230009 China

## Abstract

To deal with the growing scale of molecular systems, sophisticated modelling techniques have been designed in recent years to reduce the complexity of mathematical models. Among them, a widely used approach is delayed reaction for simplifying multistep reactions. However, recent research results suggest that a delayed reaction with constant time delay is unable to describe multistep reactions accurately. To address this issue, we propose a novel approach using state-dependent time delay to approximate multistep reactions. We first use stochastic simulations to calculate time delay arising from multistep reactions exactly. Then we design algorithms to calculate time delay based on system dynamics precisely. To demonstrate the power of proposed method, two processes of mRNA degradation are used to investigate the function of time delay in determining system dynamics. In addition, a multistep pathway of metabolic synthesis is used to explore the potential of the proposed method to simplify multistep reactions with nonlinear reaction rates. Simulation results suggest that the state-dependent time delay is a promising and accurate approach to reduce model complexity and decrease the number of unknown parameters in the models.

The advances in systems biology have raised a number of key challenges for modeling large-scale biochemical networks. Although a trend in mathematical modeling is to construct more and more mechanistically detailed models, the complexity of biological network, lack of experimental data and requirement of computing power have put a limitation on the complexity of mathematical models^1^,^2^,^3^. Recently various methods have been developed to reduce model complexity^4^,^5^. Simultaneously research works have also been conducted to explore the conditions and assumptions of these simplified models in order to obtain accurate simulations^6^,^7^. Among them, one of the important biological processes is multistep reactions that has implication in a wide range of biochemical processes, including synthesis of mRNA for a series of strands of DNA, protein synthesis when ribosome reading a series of codons of mRNA^8^, signaling transduction for the activation of a sequence of kinases from growth factor receptor to transcriptional factors^9^, degradation of polymeric carbohydrates and synthesis of metabolic^10^,^11^, cancer initiation that can be regarded as a series of gene mutations^12^, as well as telomere shortening process^13^. Although a number of mathematical models in recent years have been designed to describe these multistep pathways accurately, due to the complex nature of molecular networks, more sophisticated models are needed to simplify the multistep reaction processes.

It has been widely accepted that biochemical processes are stochastic. Recent advances in experimental technology have provided the ability to measure cellular heterogeneity in single cells^14^,^15^. Experimental studies have shown that gene expression is subject to stochastic fluctuations that lead to considerable differences in the level of expression between genetically identical cells. In addition, variation in protein levels arises from fluctuations in mRNA levels^16^. Stimulated by the pioneer work of stochastic modelling for gene expression^17^, the last ten years have seen an explosion in stochastic modelling to predict protein fluctuations in terms of the frequencies of probabilistic event^18^,^19^. Although the expression process can be modelled by a series of detailed chemical events, the model structure may be too complex to get predictive insights. To address this issue, a number of modelling techniques have been proposed to simplify the complexity of mathematical model^20^,^21^,^22^. Among them, differential equations with time delay have been used to simplify processes of multistep reactions^23^. To explore the combined effects of time delay and intrinsic noise on genetic regulation, delay stochastic simulation algorithm (delay-SSA)^24^,^25^ has been proposed to simulate discrete chemical kinetic systems. The advances in delayed modelling approaches include mathematical models for spatial effects in gene expression^26^, and stochastic reaction systems with distributed delays^27^. Other modelling techniques proposed recently include the slow-scale linear noise approximation and stochastic quasi-steady-state assumption^28^,^29^,^30^. Recently, we have proposed the memory stochastic simulation algorithm for memory reactions and a two-variable model with a new concept of length^5^,^31^.

It has been widely assumed in literature that time delay is either a constant or distributed delay with constant mean. We have used a one-step reaction model with constant, exponentially distributed or Erlang distributed delay to realize the mRNA turnover dynamics^32^. Simulation results suggest that the value of time delay may depend on the system state, rather than be a constant value. In fact, the state-dependent time delay has already been used in various research areas such as optimal control and population dynamics^33^,^34^. Although these ideas were proposed about 40 years ago, the relationship between time delay and system state remains uncertain for discrete chemical reaction settings. Recently, a model with non-constant time delay has been derived to simplify the translational process of multistep reactions^35^. In addition, research work has been conducted for exact model reduction with time delay; and closed form distribution and extension have been derived for fully bi-directional monomolecular reactions^36^,^37^. In spite of these advances, more work is still needed to address the issue of accuracy for modelling multistep reaction systems. In this work we will develop a new method using chemical reaction with state-dependent time delay to simplify multistep reaction systems accurately. The proposed method will be validated by two models for the degradation process of mRNA molecules and one model for multistep metabolic synthesis pathway.

## Results

### Mathematical model

This study considers the following system with a series of chemical reactions:


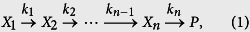


where *X*_*i*_ represents the i-th state of a molecule with copy number *x*_*i*_ and *k*_*i*_ is rate constant. Here “*P*” is the product, and it may also be “()” if it is a degradation process. Denote 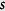 as the total copy number of molecules in all states, namely 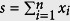. The dynamics of system (1) can be described by an ordinary differential equation (ODE) model in Supplementary Information. For simplicity, it is assumed that rate constant in each step is the same (i.e. *k*_*i*_ = *k*). Then the exact solution of this ODE model can be derived analytically. In particular, the total molecule number is represented by


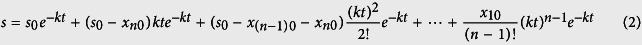


where 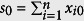. We assume that the initial condition satisfying *x*_20_ = *x*_30_ = … = *x*_*n*0_ = *y*_0_/(n − 1) with 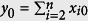. Then the total molecule number is represented by


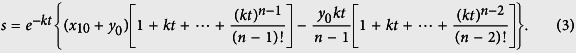


Using the Taylor polynomial and remainder term of exponential function *e*^*kt*^, the above solution is approximated by


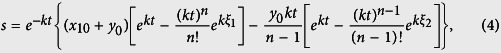


where 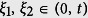 are functions of time t in the remainder terms. If the number of reactions *n* is large, such as the model of mRNA degradation in which the decay dynamics is described by a chain of eight-step, poly (A)-shortening reactions and one-step terminal deadenylation reaction (namely *n* = 9)^32^, we further assume that *ξ*_1_ = *ξ*_2_ = *ξ*.

We use a reaction with time delay to simplify the multistep process (1), which is described as follows:


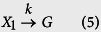



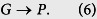


Here reaction (5) represents the first reaction of system (1), while delayed reaction (6) is a simplification of the process from state *X*_2_ to product *P*. Time delay in reaction (6) is the sum of waiting time experiencing *n* − 1 consecutive reactions from state *X*_2_ to product *P*. Thus the imaginary state G represents anyone of the intermediate states *X*_2_, 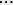, *X*_*n*_ and its molecular number is 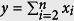. Here reactions (5) and (6) are termed as consuming and nonconsuming reactions^38^. In this work we consider systems in which the rate constants are relatively close to each other. If the multistep reaction chain involves different time-scales, a two-step reaction system will be a better approach to approximate the multistep reaction system.

### State dependent time delay

**Figure 1:**
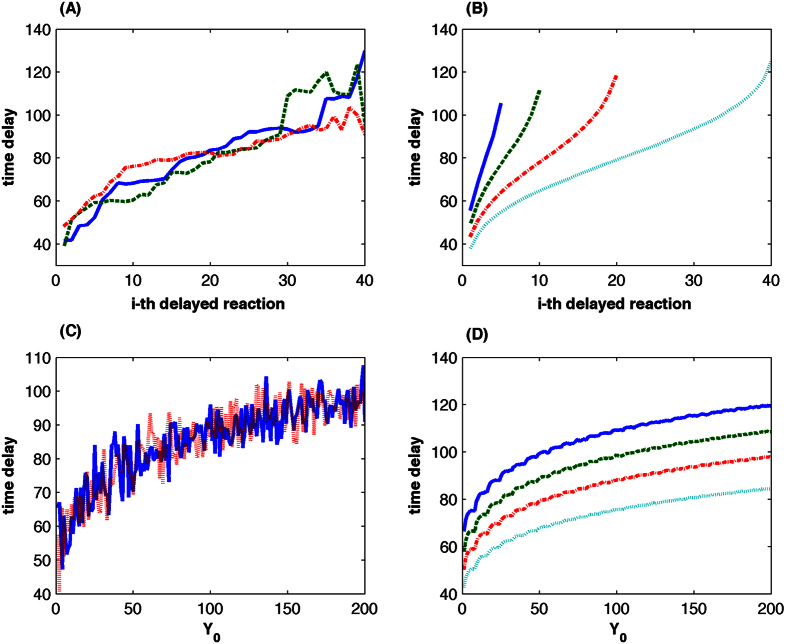
Calculated time delay using stochastic simulations of the multistep reactions process (1). (**A**) Three simulations of time delay for the decay of each molecule using initial molecular numbers *x*_10_ = 20 and y_0_ = **0**. (**B**) Averaged time delay using 1000 simulations for the decay of each molecule based on different initial number *x*_10_ but null initial imaginary species y_0_. Index *i* means the delay of the i-th molecule. (**C**) Two simulations of time delay for the decay of the first molecule using initial molecular number *x*_10_ = 20 and different values of y_0_. (**D**) Averaged time delay using 1000 simulations for the decay of the first molecule based on different values of *x*_10_ and y_0_. ((**B**,**D**): Solid-line: *x*_10_ = 5, dash-line: *x*_10_ = 10, dash-dot-line: *x*_10_ = 40, dot-line: *x*_10_ = 20).

To demonstrate the dependence of time-delay on system state, we use Supplementary Algorithm 1 to numerically calculate the value of time delay under various initial conditions. We first test the case with different values of initial molecular number *x*_10_ but fixed *y*_0_(=0). Figure 1(A) provides three stochastic simulations of time delay using *x*_10_ = 20. We also obtain 1000 stochastic simulations of time delay and present the averaged values in Fig. 1(B) based on *x*_10_ = 5, 10, 20, 40. For each initial condition, the value of time delay increases when the total molecular number decreases. The reason is that a smaller value of propensity function may lead to a larger waiting time of chemical reactions. For example, when *x*_10_ = 40, the time delay for the decay of the first molecule is t = 38.2, while that for the last molecule is t = 125.3. Similarly, if the initial molecular number *x*_10_ is larger, the delay time for the molecule of the same order is smaller. These results clearly suggest that the value of time delay depends on the values of propensity functions that are determined by the system state.

To further demonstrate the dependence of time delay on the imaginary molecule, we use Supplementary Algorithm 2 to calculate time delay for the decay of the first molecule based on different *x*_10_ and *y*_0_(>0). In this algorithm the value *y*_0_ is transferred to the initial molecule numbers (*x*_20_, *x*_30_, 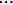 , *x*_*n*0_). It is assumed that the initial number *x*_*i*0_ satisfies *x*_20_ ≥ *x*_30_ ≥ 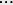 *x*_*n*0_ and the difference between these numbers is at most 1. This requirement is consistent with the assumption in the deterministic setting (*x*_20_ = *x*_30_ = 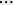 = *x*_*n*0_). For example, if *n* = 5 and *y*_0_ = 6 the initial system state is (*x*_10_, 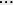 , *x*_50_) = (*x*_10_, 2, 2, 1, 1) for a given initial molecule number *x*_10_. Figure 1(C) gives two stochastic simulations of time delay for the delay of the first molecule based on *x*_10_ = 20 but different values of *y*_0_.The averaged value of time delay in Fig. 1(D) using 1000 stochastic simulations suggests that time delay also depends on the number of imaginary species in the system.

### Formula for calculating time delay

**Figure 2:**
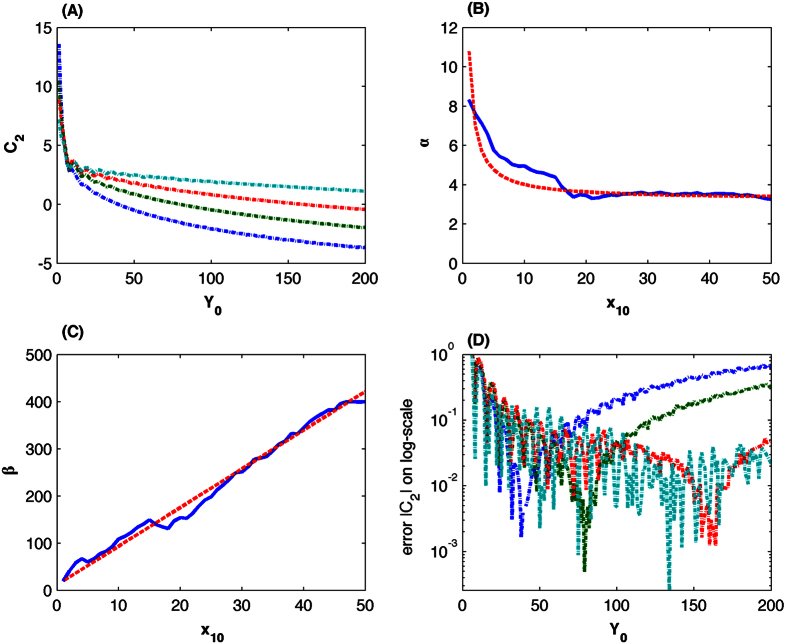
Algorithm for calculating time delay that is dependent on system state. (**A**) Estimated optimal values of *C*_2_ based on different system states (*x*_10_, *y*_0_) that match time delay showing in Fig. 1(D). (Solid-line: *x*_10_ = 5, dash-line: *x*_10_ = 10, dash-dot line: *x*_10_ = 20, dot-line: *x*_10_ = 50). (**B**) Estimated values of *α* based on different values of *x*_10_. (Solid line: estimated values based on simulated time delay in Fig. 1(B); dash line: *α* = 3.25 + 7.5/*x*_1_). (**C**) Estimated values of *β* based on different values of *x*_10_. (Solid line: estimated values based on simulated time delay in Fig. 1(B); dash line: *β* = 11.8 + 8.2*x*_1_). (**D**) Difference between the predicted values of *C*_2_ and optimal values of *C*_2_ in Fig. 2(A) on a logarithmic scale. (Solid-line: *x*_10_ = 10, dash-line: *x*_10_ = 10, dash-dot line: *x*_10_ = 20, dot-line: *x*_10_ = 50).

Methods section derives a formula for calculating time delay based on a given system state. However, an unsolved question is the value of *C*_2_ in (25) explicitly includes time t. Here we find an approximation of *C*_2_ through numerical computation. We first search for the optimal value of *C*_2_ for different values of *x*_10_ and *y*_0_ using the derived expression (25) to match the calculated time delay in Fig. 1(D). Note that this computation is based on different values of *x*_10_(=5, 10, …, 100) but Fig. 2(A) only shows the results for 4 values of *x*_10_. Each line in Fig. 2(A) represents the optimal value of *C*_2_ for a particular value of *x*_10_ but different values of *y*_0_. For a fixed value of *y*_0_, the smaller the value of *x*_10_ is, the smaller the value of *C*_2_ becomes. The optimal value of *C*_2_ in Fig. 2(A) suggests that it is a monotonically decreasing function of *y*_0_. In addition, the value of *C*_2_ is −1/*y* when *y* = *x*_1_ = (*n* − 1). Thus it is assumed that


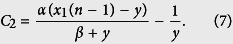


To determine the values of *α* and *β*, we further estimate these values by matching the determined time delay using equation (25) with those shown in Fig. 1(D). The estimated values in Fig. 2(B,C) suggest that the values of *α* and *β* may also be functions of *x*_1_. Based on the values in Fig. 2(B,C), we use the following two functions to approximate *α* and *β*, namely *α* = 3.25 + 7.5/*x*_1_ and *β* = 11.8 + 8.2*x*_1_. Thus the final expression of the approximated *C*_2_ is


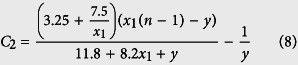


To validate the proposed approach (8), we compare the optimal value of *C*_2_ in Fig. 2(A) with that determined by (8). Figure 2(D) shows the difference between these two values under different values of *x*_1_ and *y* on a logarithmic scale. The optimal value of *C*_2_ for small value of *y*_0_ in Fig. 2(A) is larger than that for large value of *y*_0_. The estimated value of *α* from *α* = 3.25 + 7.5/*x*_1_ in Fig. 2(B) matches the optimal value of *α* very well only for large value of *y*_0_. Thus, when *y*_0_ is small, the error of *C*_2_ is relatively large, but it is still quite small, which suggests that approach (8) provides accurate approximation to the optimal value of *C*_2_.

### Time delay model for mRNA degradation

The degradation process of mRNA molecules is a typical multistep reactions system. In experimental studies, a large sample of cells are genetically modified or treated with inhibitors to stop transcription and thus kinetic information of a decaying mRNA species can be obtained^39^. Recently single-cell and single-molecule techniques have advanced our understanding of mRNA turnover^40^. The accuracy of decay measurement varies with the technique used^41^,^42^,^43^. A detailed mechanistic model has been designed to describe the degradation process exactly^44^,^45^. In addition, a simplified model of multistep reactions was proposed by combining a number of terminal deadenylation reactions into a single reaction^32^. However, it is difficult to derive accurate information of half-life from detailed mechanistic models.

Next we apply our state-dependent delay model to study the mRNA degradation process of gene ribosomal protein L30 (*RPL30*). Experimental studies have demonstrated the transcript decay dynamics of two constructs for this gene, namely construct A-ACT1 UAS (upstream activating sequence) and construct B-RPL30 UAS^46^. In experiments, mRNA molecule decaying dynamics was monitored after blocking transcription by using drug 1,10-phenanthroline^46^. Therefore, it is assumed that no further transcription occurs after drug application. Since there is no explicit information regarding the mRNA copy number, we test the case with initial total mRNA number *s*_0_(=100).

Here we use the delayed reactions (5, 6) to represent the degradation dynamics, where *X*_1_ is mRNA molecule with full length of poly(A)-tail and imaginary species G represents transcripts in the poly(A)-shortening process. The initial number of imaginary species *y*_0_ and degradation rate *k* are unknown parameters that will be estimated to match experimental data. In addition, the manifesting time of these initial imaginary species is uniformly distributed in time interval [0, MT] and


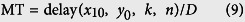


where delay(*x*_10_, *y*_0_, *k*, *n*) is the time delay determined by the initial system state (*x*_10_, *y*_0_), degradation rate *k*, and number of steps *n* in system (1). For initial imaginary species, a few reactions of the multistep reactions may have already taken place, and the time to reach the product should be smaller than the calculated time delay delay (*x*_10_, *y*_0_, *k*, *n*). Thus we use a factor (D > 1) to adjust the time delay of initial imaginary species. We use an Approximate Bayesian Computation (ABC) rejection sampling algorithm^47^ to search for optimal parameters of *y*_0_*, k* and *D*. The time delay of each delayed reaction is calculated based on the current system state (*x*_1_, *y*). We select 150 sets of the inferred model parameters with smaller error and use the set with minimal error as our final estimation.

**Figure 3:**
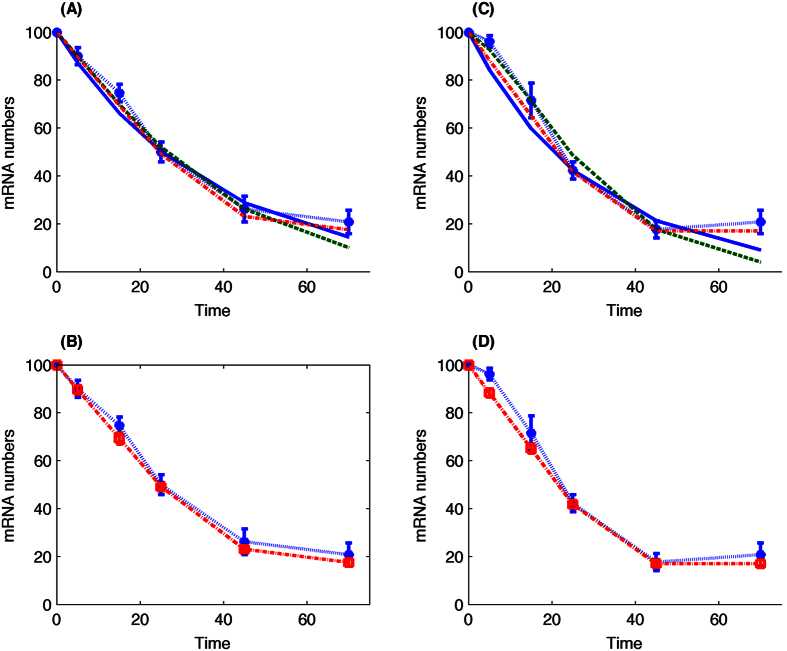
Simulation of mRNA degradation for gene *RPL30* using the state-dependent delay model. Numerical results are the averaged molecular numbers using 1000 stochastic simulations. (**A**,**B**) Construct ACT1 using estimated parameters *k* = 0.1260, *y*_0_ = 23, *D* = 1.7184. (**C**,**D**) Construct RPL30 using estimated parameters *k* = 0.1260, *y*_0_ = 17, *D* = 1.7525. (**A**,**C**: Star-dash line with error bar: experimental data. Solid-line: one-step model; dash-line: two-variable model. dash-dot line: time-dependent delay model). (**B**,**D**: star-dash line with error bar: experimental data, square-dash line with error bar: time-dependent delay model).

Based on 1000 simulations, Fig. 3 shows that the state-dependent delay model is able to provide accurate description of mRNA degradation dynamics for the two constructs of gene *RPL30*. We also present simulation results of the one-step model and two-variable model that were shown in ref. 5. For each model, we use absolute error to measure the difference between simulation and experimental data. In addition, we provide error bars of experimental data and count the number of observation time points for each simulation that is beyond the error bar. Compared with the one-step and two-variable models, STable 1 suggests that this new model with state-dependent delay provides more accurate simulations to the experimental data. Distributions of inferred parameters in SFigure 1(A and D) suggest that ~25% of initial mRNA molecules are imaginary species, namely the transcripts in the poly(A)-shortening process. In addition, distributions of value D in SFigure 1(B and F) suggest that the degradation time points of these shortened transcripts are distributed in an interval that is ~60% of the normal time delay interval. Thus these imaginary species may already exist in the middle of the shortening process.

Simulation results in Fig. 3 are based on the assumption that the initial total transcripts number is *s*_0_ = 100. The next question is whether this assumed initial total mRNA number influences the estimated model parameters. To answer this question, we simulate the delay model using the estimated parameters and different initial total mRNA *s*_0_[=10, 50, 150, 200]. The rate constant k and parameter D remain unchanged, but the value of *y*_0_ is rescaled according to *s*_0_ to maintain a fixed ratio *y*_0_/*s*_0_. Simulation results in SFigure 2 suggest that our estimated parameters can also produce accurate simulations for various initial mRNA numbers. The difference between simulation and experimental data is relatively large when the initial total MRNA number is small.

### Time delay model in gene expression

The success of our proposed method for simplifying a multistep reaction system leads to the next study to model the expression of a cell cycle-regulated gene (e.g. *SWI5*) based on the measured change in the mRNA turnover during a cell cycle^48^. SWI5p is a transcription regulator of late mitosis genes and its expression is tightly regulated during the cell cycle. It was measured to degrade with 8 min half-lives^43^. In addition, *NDD1* (nuclear division defective) is an essential gene for the expression of gene *SWI5*. It has been shown that overexpression of *NDD1* enhances the expression of SWI5^49^. The expression of gene NDD1 peaks during the S phase and is essential for the expression of its target gene *SWI5* during the G2/M phase^49^,^50^.

A simple mathematical model has been proposed to describe the expression of gene *SWI5* based on experimental data measured in single cells. In this model the degradation of mRNA molecules is described by a one-step reaction and simulation is used to measure the half-life of mRNA molecules^48^. To accurately measure the half-life of mRNA transcripts, we propose a delayed model to describe the expression of gene *SWI5*. It is assumed that the transcription of this gene is activated by TF NDD1 with transcriptional rate


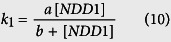


where *a* and *b* are parameters for genetic regulation. In addition, the elongation process needs time for RNAP II polymerase travelling along the template DNA. Due to the fixed number of DNA in a single cell, it is assumed that the time of elongation process is relatively fixed and a delay reaction with constant time delay is used to represent the synthesis of mRNA transcripts. Then mRNA transcripts translocate from nucleus to cytosol, which is also modelled by a delay reaction with constant time delay for simplicity. Finally mRNA molecules in cytosol decay via a multistep process that is simplified as a state-dependent delay reaction (5, 6). Thus the proposed model for the expression of gene *SWI5* is given below:


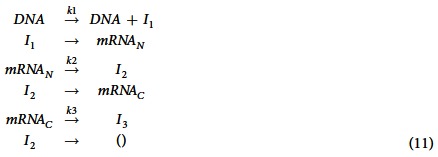


where *mRNA*_*N*_ and *mRNA*_*C*_ are mRNA molecules in nucleus and cytosol; *I*_1_, *I*_2_, and *I*_3_ are imaginary species for *mRNA*_*N*_, *mRNA*_*C*_, and shortening mRNA, respectively. We use the inferred concentration of [NDD1] in ref. 51 as the activity of this TF, which is consistent with the drafted TF activity in ref. 48. In addition, experimental studies show that gene expression is regulated by mechanisms of cell cycle. In yeast, the mitosis process at ~49 min of each cell cycle terminates the process of transcription^52^. This regulatory mechanism is realized by the assumption that the activity of [NDD1] is zero after 49 min of each cell cycle.

**Figure 4:**
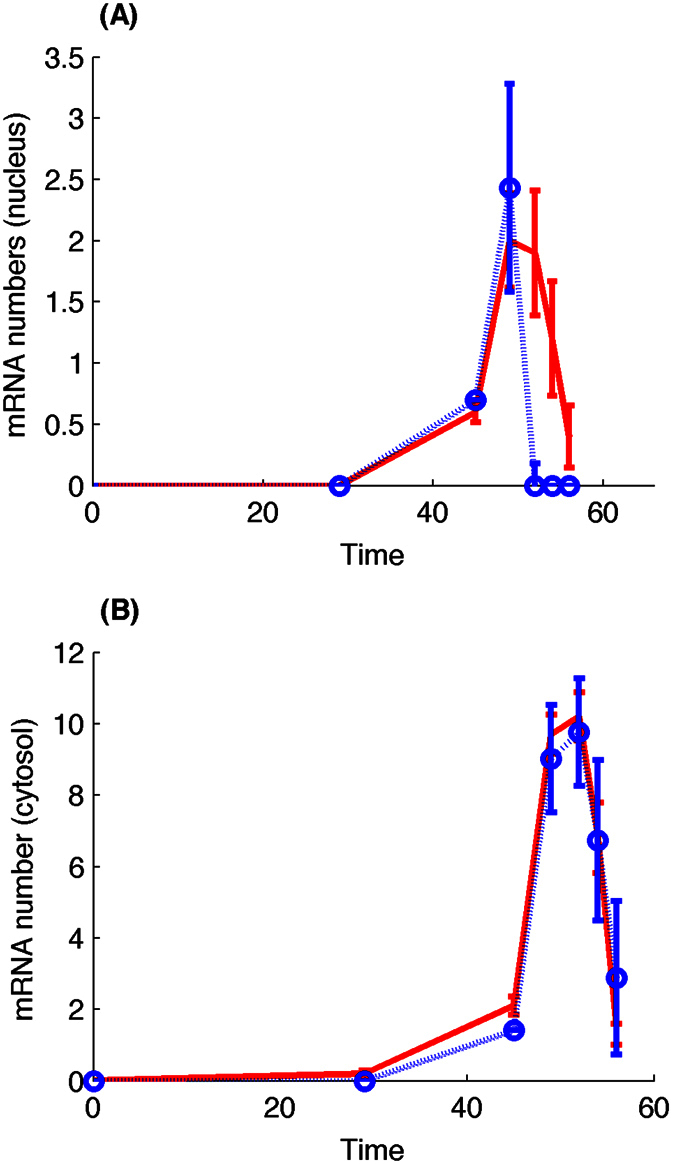
Simulation of gene transcription for gene *SWI5* using the state-dependent delay model. Numerical results are the averaged molecular numbers using 1000 stochastic simulations. (**A**) mRNA copy number in nucleus. (**B**) mRNA copy number in cytosol. (dot line: experimental data; solid line: simulations). Estimated parameters are *a* = 9.148, *b* = 3.390, *τ*_1_ = 46.665, *τ*_2_ = 0.733, *k*_2_ = 2906.04, *k*_3_ = 1.297.

The measured mRNA copy numbers in single cells^48^ are used to infer regulation parameters *a*, *b*, rate constant *k*_3_, and transcription and translocation delays. We use the ABC rejection sampling algorithm to search for optimal model parameters. Using simulation error to both cytosol and nucleus data as the criterion, we select 150 sets of model parameters with smaller simulation error. The parameter set with the minimal error is the final inference result. Figure 4 shows that the numerical simulation matches experimental data very well. In addition, the distribution of transcriptional time delay in SFigure 3(A) is consistent with the experimental estimations showing that the time delay in transcription is ~35 min^53^. An interesting observation is the degradation rate of mRNA is *k*_3_ = ~1.29/min.

### Multistep pathway of metabolic synthesis

The previous two systems are used to study the accuracy of state-dependent time delay for simplifying multistep reaction processes with a fixed rate constant. The next question is whether the state-dependent time delay is able to approximate multistep reactions with varied reaction rates with good accuracy. Here we discuss a system that is a simplified representation of the pathway for aliphatic glucosinolate biosynthesis^11^. This system considers the chain elongation process as a series of sequential events. Each chain elongation cycle is simplified into a one step reaction *E*_*i*_ → *E*_*i*+1_. Here we only consider multistep reactions in wild-type cells and thus the process for reducing the conversion of chain elongated 2-oxo acides into final glucosinolates is not included in this model^11^. This pathway contains six reactions


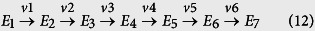


where *E*_1_ is the input and *E*_7_ the product. In each step the reaction rate is a Michaelis-Menten function


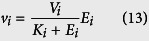


where *V*_*i*_ and *K*_*i*_ are the maximal synthesis rate and equilibrium constant, respectively. The detailed values of these parameters in terms of concentrations are given in ref. 11. Since a plant cell stretches from 10 to 100 micrometers, we assume that the size of a plant cell is 4*10^−14^ liter. Thus a concentration of 1 *μM* is about 25600 molecules^54^. The values of *V*_*i*_ and *K*_*i*_ are converted into those with unit of molecular numbers as (*V*_1_, …, *V*_6_) = (37.07, 38.27, 73.44, 35.84, 9.31, 2.08) and (*K*_1_, …, *K*_6_) = (23859200, 12185600, 11852800, 9164800, 6476800, 2073600). For this process of six-step reactions, we use a delayed reaction to simplify the model. Here the first reaction 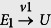 remains unchanged. For the delayed reaction *U* → *E*_7_, we use (25) to calculate time delay using reaction rate


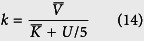


where U/5 represents the averaged molecular number of *E*_*i*_ (i = 2 ~ 6). We use 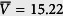 and 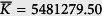 are the harmonic mean of parameters 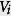 and *K*_*i*_ (i = 2 ~ 6), respectively. For example, the value of 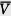 is given by


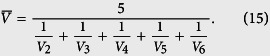


**Figure 5:**
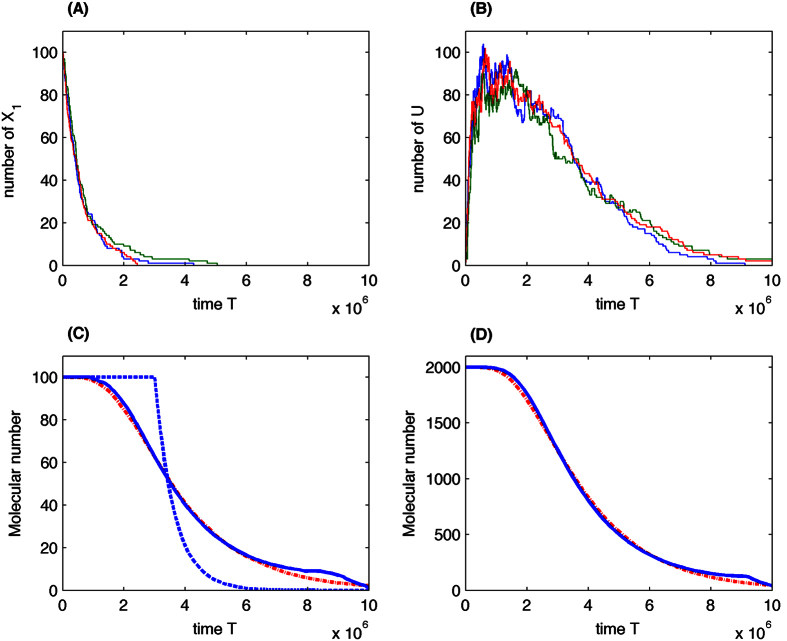
Simulation of substrate competition of metabolic pathway. (**A**) Three stochastic simulations of *E*_1_. (**B**) Three stochastic simulations of 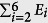. (**C**) Simulations of metabolic pathway with initial condition *E*_1_ = 100 (Solid-line: multistep reaction pathway; dash-dot line: delayed reaction with state-dependent time delay; dish-line: delayed reaction with constant time delay). (**D**) Simulations of metabolic pathway with initial condition *E*_1_ = 2000 (Solid-line: multistep reaction pathway; dash-dot line: delayed reaction with state-dependent time delay).

Figure 5 gives three stochastic simulations of the multistep pathway using the SSA and initial condition *E*_1_ = 100 and *E*_*i*_ = (i = 2 ~ 6}. Simulated values of *E*_1_ in Fig. 5(A) show that the molecules are converted into molecules *E*_2_ soon but the molecular number of 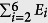 in Fig. 5(B) stays in the high level for quite a long time. Figure 5(C) gives the averaged total molecule number 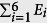 over 1000 simulation using the multistep pathway and state-dependent time delay. Numerical results suggested that the state-dependent time delay reaction approximates the multistep reactions accurately. Simulations with an initial molecular number *E*_1_ = 2000 in Fig. 5(D) confirm this result. We have also tested the accuracy of delayed reaction with constant time delay. Simulation in Fig. 5(C) using constant delay (=3000000) suggests that the total molecular numbers stays at 100 before t = 3000000 and then decreases much quicker than that obtained by using state-dependent time delay. Simulations suggest that it is not appropriate to use constant time delay reaction to approximate this test system.

## Conclusions

In this work, we propose a new algorithm to calculate time delay in chemical reaction systems according to the system state. Using the process of multistep reaction systems as the test problem, we utilize both analytical solution of ODE model and stochastic simulation of chemical reaction systems to determine the relationship between the system state and value of time delay. The proposed method is applied to model the degradation process of mRNA molecules based on experimental data measured in single cells and a multistep pathway for metabolic synthesis. For the first test system of mRNA degradation, our model gives simulations with better accuracy than those of the existing models. For the second test system of gene expression, our model provides simulated dynamics with very good accuracy for both synthesis and degradation of mRNA transcripts. Simulation of the third system suggests that the state-dependent time-delay can be applied to approximate multistep reactions with nonlinear reaction rates with very good accuracy. Simulation results in this work suggest that the proposed method is an effective approach to approximate multistep reaction systems more accurately. Compared with the full multistep reaction model, it is also an efficient approach to reduce computing time of stochastic simulation, save computer storage, and decrease the number of unknown parameters that should be estimated from experimental data.

Half-life is an important concept to measure the degradation process in biological studies. It is the time required for the amount of a species to fall to a half of the initial value. Based on the widely used assumption that the quantity follows an exponential decay, the decay rate constant can be determined by the half-life value or vice versa. However, for many biological molecules such as mRNA transcripts, the decaying dynamics may not follow an exponential process; rather it may be an event of multistep reactions. Thus, molecules in the intermediate states may also be important to determine the value of half-life. This may be a reason to explain the difference between the determined half-life values under different experimental conditions. Using the inferred degradation rate in the state-dependent delay model, our results suggest that more work is needed to establish the relationship between half-life and degradation rate constant of biological species.

This work is based on the assumption that rate constants in the multistep reactions are the same. However, rate constants in biological systems are usually different from each other. We have conducted further computation for testing the influence of parameter variation on the simulation accuracy. Numerical results suggested that the difference between simulation using the same rate constant and that using different rate constants is proportional to the variance of rate constants. Thus, the proposed method with state-dependent time delay is applicable to model pathways in which the rate constants are relatively close to each other, such as the three systems discussed in this work. However, if the multistep reaction chain involves different time-scales, namely the difference between the rate constants is large, we may need to use multiscale approaches by dividing the pathway into two or more subsystems. Then a two-step delayed reaction system may be a better approach to approximate the multistep reaction system.

Using the multistep reaction system as the test system, this work represents a further step forward in developing accurate delayed models for chemical reaction systems. However, more research work is still strongly needed to study other types of multistep reaction systems as well as the complex systems that include multistep reactions processes as subsystems. For genetic regulation, for example, it would be important to study TF regulation by including DNA/mRNA/protein interactions and also explore the mechanisms of transcriptional elongation. In addition, the proposed approach is based on the mass action law kinetics. Delay models based on other approaches, such as the Hill function for catalyzing enzyme kinetics or Shea-Ackers model for genetic regulation, would also be interesting research problems. Another significant challenge is the possible large variations of estimated parameter values that all can faithfully realize experimental data, in particular for inferring unknown parameters in stochastic models. Currently a number of approaches use important system properties (e.g. robustness property) as additional criteria to select estimation candidature^2^,^55^. More system properties and research work are needed to address this issue. All these interesting problems will be potential topics of future research.

## Methods

### State-dependent time delay

For delayed model (5,6), we need to determine the value of time delay based on current system state (*X*_1_,*G*) with molecule numbers (*x*_1_, *y*). When the first reaction fires, a molecule of *X*_1_ moves into the queue structure of time delay *L* in which there are already *y* imaginary molecules. When the newly added molecule turns to product *P*, it is assumed that all *y* molecules queued before the newly added molecule already turn into the product. Thus, when the first molecule from *X*_1_ state turns to product, the total molecule number should be reduced from *x*_1_ + *y* to *x*_1_ − 1. The time required for this process, namely time delay, is defined as


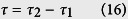


where *τ*_1_ is the firing time of the first reaction *X*_1_ → *X*_2_, and *τ*_2_ is the firing time of the last reaction *X*_*n*_ → *P* to let the system state be *s* = *x*_1_ − 1.

We use computational simulations to determine the value of time delay. Here *τ*_1_ is determined by the stochastic simulation algorithm (SSA)^56^. The key issue is how to determine the value of *τ*_2_. Given a system state (*x*_1_, *y*) at time *t*, the time *τ*_2_ for the first *X*_1_ molecule turns into product *P* is


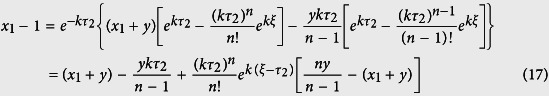


which can be simplified as


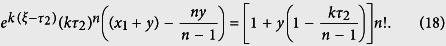


Denote *C*_1_ = *x*_1_ + *y* − *ny*/(*n* − 1), *C*_2_ = 1 − *kτ*_2_/(n − 1), *C* = (1 + *C*_2_*y*)*n*!/*C*_1_, the above equation is simplified as


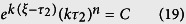


There are a number of undetermined coefficients in the above equation. Thus, we first use a special case to determine the value of *ξ* by letting *y* = 0. In this case, *C*_1_ = *x*_1_, *C* = *n*!/*C*_1_. We rewrite the above equation as


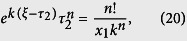


Here 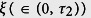
is a function of *τ*_2_. To determine the optimal value of *ξ*, we compared the time delays obtained by using a number of values [*ξ* = (0, 0.1, …, 1)*τ*_2_] in (20) with those obtained from stochastic simulations. We found that, when *ξ* = *τ*_2_/2, formula (20) provides more accurate estimate for time delay than other values. In this case, it becomes


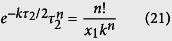


Using the Lambert *W* function, the solution of *τ*_2_ is given by


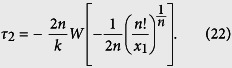


Next we return to the general case when *y* > 0. When *C*_1_ = 0, the left hand side of equation (18) is zero. By letting the right-hand side be zero, we have that


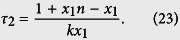


When *C*_1_ ≠ 0, using the optimal value 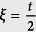, the time to reach the system state with *x*_1_ − 1 molecules is


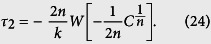


In summary, we have the following expression for calculating time delay *τ* = *τ*_2_ − *τ*_1_, where the value of *τ*_1_ is determined by the SSA, and


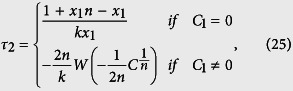


Note that *C*_2_ is dependent on the values of *x*_1_, *y*, and time *t*, which is determined in Results section by numerical simulations.

### SSA with state-dependent time delay

This work proposes the following modelling framework with state-dependent time delay to simplify multistep reaction events. Here we need to simulate a well-stirred mixture of *N*(≥1) molecular species 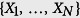 that chemically interact inside some fixed volume Ω at a constant temperature and through *M* reaction channels 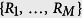, which includes *M*_1_ elementary reactions and *M*_2_ delayed reactions (*M* = *M*_1_ + *M*_2_). Here a delayed reaction may be a reaction with constant time delay, distributed delay that follows a distribution, or state-dependent time delay that is simplified from the lumped multistep chemical reactions (1). The system state is denoted as 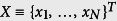 where *x*_*i*_(*t*) is the copy number of species *X*_*i*_. For each delayed reaction, we define an imaginary species *G*_*i*_ to represent the corresponding intermediate species. We also define a stoichiometric vector ν_*j*_ for non-delayed reactions, as well as consuming and manifesting stoichiometric vectors ν_*j*_ and *u*_*j*_ for delayed reactions (5) and (6), respectively. For each reaction channel, a propensity function *a*_*j*_(*X*) is defined and *a*_*j*_(*X*)*dt* represents the probability that this reaction will fire inside Ω in the next infinitesimal time interval [*t*, *t* + *dt*]. Detailed algorithm is given below.

**Algorithm:** State-dependent Delay SSA (SD-SSA)

Set initial molecular numbers at *t* = 0, and an empty queue structure *L* for storing the information of delayed reactions.

Step 1. Calculate propensity functions *a*_*i*_(*X*), 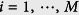 and 
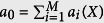
.

Step 2. Generate a uniform random number 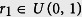 and determine the waiting time *μ* of next reaction by


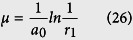


where *r*_1_ ~ *U*(0,1).

Step 3. Compare *μ* with the least update time *δ*_*min*_ in the queue structure *L* to check whether there is any delayed reaction that is scheduled to finish within [*t*, *t* + *μ*).

Step 4. IF*δ*_*min*_ < *μ* (update the delayed reaction with index k at *δ*_*min*_)


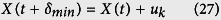


ELSE: Generate a sample *r*_2_ ~ *U*(0,1) to determine the index *j* of next reaction


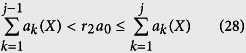


update the system state by


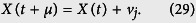


If reaction with index j is a delayed reaction, use the constant delay; or generate a sample for the distributed delay reaction; or use (25) to calculate the delay value *τ* if *R*_*j*_ is a reaction with state-dependent time delay. Then add index *j* and update time *δ* = *t* + *μ* + *τ* to the queue structure *L*.

Step 5. Go to Step 2.

Note that this algorithm is based on the so-called rejection delay-SSA^57^. A more precise algorithm can be developed if we consider the change of propensity functions due to the update of a delayed reaction in step 2^58^. In addition, the calculated value of *τ*_2_ is deterministic but the value of time delay in Fig. 1(A,C) is stochastic. Similar to other approaches using distributed delay^59^, we can generate random samples of *τ*_2_ from a random variable whose mean is the calculated deterministic value.

## Additional Information

**How to cite this article**: Wu, Q. and Tian, T. Stochastic modeling of biochemical systems with multistep reactions using state-dependent time delay. *Sci. Rep.* **6**, 31909; doi: 10.1038/srep31909 (2016).

## Supplementary Material

Supplementary Information
