# Supplementary material for: Stochastic modeling of biochemical systems with multistep reactions using state-dependent time delay
Source: Sci Rep. 2016 Aug 24;6:31909. doi: 10.1038/srep31909 (PMC4995396; doi:10.1038/srep31909)
Supplement: Supplementary Information [file srep31909-s1.pdf]

Supplementary Information

Stochastic modeling of biochemical systems with multistep reactions using state-dependent time delay

Qianqian Wu and Tianhai Tian

This supplementary information gives a detailed description of the exact solution of the multi-step chemical reactions in Section 1. Then Section 2 derives the formula to calculate time delay based on the analytical solution of the ordinary differential equation (ODE) model for the multi-step reaction system. Section 3 gives two algorithms for calculating time delay exactly using the Stochastic Simulation Algorithm (SSA). Finally we give three supplementary figures for additional simulation results.

Section 1 Multi-step chemical reactions system

The starting point of this study is the following system with a series of chemical reactions:

$$X_1 \xrightarrow{k_1} X_2 \xrightarrow{k_2} \ldots \xrightarrow{k_{n-1}} X_n \xrightarrow{k_n} P \quad X_1 \overset{k_1}{\rightarrow} X_2 \overset{k_2}{\rightarrow} \cdots \xrightarrow{k_{n-1}} X_n \overset{k_n}{\rightarrow} P \qquad (1)$$

where X_i X_i represents the i-th state of a molecule with copy number x_i x_i and k_i k_i is rate constant. Here " P P" is the product, and it may also be "( )" if it is a degradation process. Denote s s as the total copy number of molecules in all states, namely $s=\sum_{i=1}^{n} x_i$ $s = \sum_{i=1}^{n} x_i$. The dynamics of system (1) can be described by an ODE model, given by

$$\begin{aligned} \frac{dx_1}{dt} &= -k_1 x_1 \\ \frac{dx_2}{dt} &= k_1 x_1 - k_2 x_2 \\ &\vdots \\ \frac{dx_n}{dt} &= k_{n-1} x_{n-1} - k_n x_n \end{aligned} \qquad . \qquad (2)$$

For simplicity, it is assumed that $k_1 = k_2 = \ldots = k_n = k$.$k_1 = k_2 = \cdots k_n = k$ The exact solutions of system (2) can be derived as

$$\begin{aligned} x_1 &= x_{10} e^{-kt} \\ x_2 &= x_{10} kte^{-kt} + x_{20} e^{-kt} \\ x_3 &= \frac{x_{10}}{2} k^2 t^2 e^{-kt} + x_{20} kte^{-kt} + x_{30} e^{-kt} \\ &\vdots \\ x_n &= \left[\frac{x_{10}}{(n-1)!}(kt)^{n-1} + \frac{x_{20}}{(n-2)!}(kt)^{n-2} + \cdots + x_{n0} \right] e^{-kt} \end{aligned} \qquad , \qquad (3)$$

where x_{i0} x_{i0} represents the initial copy number of $\mathrm{X_i}$ at t=0. Therefore, the total molecule number is represented by

$$s = s_0 e^{-kt} + (s_0 - x_{n0}) kte^{-kt} + (s_0 - x_{(n-1)0} - x_{n0}) \frac{(kt)^2}{2!} e^{-kt} + \cdots + \frac{x_{10}}{(n-1)!}(kt)^{n-1} e^{-kt} . \qquad (4)$$

where $s_0 = (x_{10} + \ldots + x_{n0})$ $s_0 = (x_{10} + \cdots + x_{n0})$. We assume that the initial conditions satisfy

$$x_{20} = x_{30} = \cdots = x_{n0} = \frac{y_0}{n-1}. \quad (5)$$

Then the total molecule number is represented by

$$s = e^{-kt}\left\{(x_{10}+y_0)\left[1+kt+\cdots+\frac{(kt)^{n-1}}{(n-1)!}\right] - \frac{y_0 kt}{n-1}\left[1+kt+\cdots+\frac{(kt)^{n-2}}{(n-2)!}\right]\right\}. \quad (6)$$

Using the Taylor polynomial and remainder term of exponential function e^{kt} e^{kt}, given by

$$e^{kt} = 1+kt+\cdots+\frac{(kt)^{n-1}}{(n-1)!}+\frac{(kt)^n}{n!}e^{k\xi_1} \quad (7)$$

the above solution is approximated by

$$s = e^{-kt}\left\{(x_{10}+y_0)\left[e^{kt}-\frac{(kt)^n}{n!}e^{k\xi_1}\right] - \frac{y_0 kt}{n-1}\left[e^{kt}-\frac{(kt)^{n-1}}{(n-1)!}e^{k\xi_2}\right]\right\}. \quad (8)$$

where $0 \le \xi_1, \xi_2 \le t$ are unknown in the remainder term, whose values are functions of time t . If the number of reactions n is large, such as the model of mRNA degradation in which the decay dynamics is described by a chain of eight-step poly (A)-shortening reactions and one-step terminal deadenylation reaction (namely nn=9) [1], we further assume that $\xi_1 = \xi_2 = \xi$ $\xi_1 = \xi_2 = \xi$.

Section 2. Formulation of time delay

We use a reaction with time delay to simplify the multi-step process (1), which is described as follows:

$$X_1 \xrightarrow{k} G \; X_1 \overset{k}{\rightarrow} G, \quad (9)$$

$$G \longrightarrow P \; G \rightarrow P. \quad (10)$$

Here reaction (9) represents the first reaction of system (1), while delayed reaction (10) is a simplification of the process from state X_2 X_2 to product P P. Time delay in reaction (10) is the sum of waiting time experiencing n-1 consecutive reactions from state X_2 X_2 to product P P. Thus the imaginary state G represents anyone of the intermediate states $X_2,\ldots,X_n$ $X_2,\cdots,X_n$and its molecular number is $y=\sum_{i=1}^{n} x_i$ $y = \sum_{i=2}^{n} x_i$. Here reactions (9) and (10) are termed as consuming and nonconsuming reactions [2]. In this work we consider systems in which the rate constants are relatively close to each other. If the multi-step reaction chain involves different time-scales, a two-step reaction system will be a better approach to approximate the multi-step reaction system.

We need to determine the value of time delay based on current system state (X_1,G X_1, G) with molecule numbers (x_1,y) (x_1,y). When the first reaction fires, a molecule of X_1 X_1 moves into the queue structure of time delay L L in which there are already y imaginary molecules. When the newly added molecule turns to product P, it is assumed that all y molecules queued before the newly added molecule already turns into the product. Thus, when the first molecule from X_1 X_1 state turns to product, the total molecule number should be reduced from x_1+y x_1+y to x_1-1 x_1-1. The time required for this process, namely time delay, is defined as

$$\tau = \tau_2 - \tau_1 \quad (11)$$

where τ_1 τ_1 is the firing time of the first reaction $X_1 \rightarrow X_2$, and τ_2 τ_2 is the firing time of the last reaction $X_n \rightarrow P$ to let the system state be $s = x_1 - 1$ $s = x_1 - 1$.

We use computational simulations to determine the value of time delay. Here τ_1 is determined by the stochastic simulation algorithm [3]. The key issue is how to determine the value of τ_2. According to (3) with $\xi_1 = \xi_2 = \xi$, we have that

$$s = e^{-kt}\left\{(x_{10}+y_0)\left[e^{kt} - \frac{(kt)^n}{n!}e^{k\xi_1}\right] - \frac{y_0 kt}{n-1}\left[e^{kt} - \frac{(kt)^{n-1}}{(n-1)!}e^{k\xi_2}\right]\right\} \tag{12}$$

Given the system state as (x_1, y) at time t, the time τ_2 for the first X_1 molecule turns into product P is, namely the total molecular number is $s = x_1$-1,

$$\begin{aligned} x_1 - 1 &= e^{-k\tau_2}\left\{(x_1+y)\left[e^{k\tau_2} - \frac{(k\tau_2)^n}{n!}e^{k\xi}\right] - \frac{yk\tau_2}{n-1}\left[e^{k\tau_2} - \frac{(k\tau_2)^{n-1}}{(n-1)!}e^{k\xi}\right]\right\} \\ &= (x_1+y) - \frac{yk\tau_2}{n-1} + \frac{yk\tau_2}{n-1}\frac{(k\tau_2)^{n-1}}{(n-1)!}e^{k(\xi-\tau_2)} - (x_1+y)\frac{(k\tau_2)^n}{n!}e^{k(\xi-\tau_2)} \\ &= (x_1+y) - \frac{yk\tau_2}{n-1} + \frac{yn}{n-1}\frac{(k\tau_2)^n}{(n)!}e^{k(\xi-\tau_2)} - (x_1+y)\frac{(k\tau_2)^n}{n!}e^{k(\xi-\tau_2)} \\ &= (x_1+y) - \frac{yk\tau_2}{n-1} + \frac{(k\tau_2)^n}{n!}e^{k(\xi-\tau_2)}\left[\frac{ny}{n-1} - (x_1+y)\right] \end{aligned}, \tag{13}$$

which can be simplified as

$$\begin{aligned} &\frac{yk\tau_2}{n-1} - 1 - y = \frac{(k\tau_2)^n}{n!}e^{k(\xi-\tau_2)}\left[\frac{ny}{n-1} - (x_1+y)\right] \\ &1 + y - \frac{yk\tau_2}{n-1} = \frac{(k\tau_2)^n}{n!}e^{k(\xi-\tau_2)}\left[(x_1+y) - \frac{ny}{n-1}\right] \\ &1 + y\left(1 - \frac{k\tau_2}{n-1}\right) = \frac{(k\tau_2)^n}{n!}e^{k(\xi-\tau_2)}\left[(x_1+y) - \frac{ny}{n-1}\right] \\ &(k\tau_2)^n e^{k(\xi-\tau_2)}\left[(x_1+y) - \frac{ny}{n-1}\right] = \left[1 + y\left(1 - \frac{k\tau_2}{n-1}\right)\right]n! \end{aligned}. \tag{14}$$

Denote

$$\begin{aligned} C_1 &= x_1 + y - \frac{ny}{n-1}, \\ C_2 &= 1 - \frac{k\tau_2}{n-1}. \\ C &= \frac{1 + C_2 y}{C_1}n!, \end{aligned} \tag{15}$$

Equation (14) is simplified as

$$e^{k(\xi-\tau_2)}(k\tau_2)^n = C\,. \tag{16}$$

There are a number of undetermined coefficients in (16). Thus, we first use a special case to determine the value of ξ by letting $y=0$ $y = 0$. In this case, $C_1 = x_1, C = n!/C_1$. We rewrite Eq. (16) as

$$e^{k(\xi-\tau_2)}\tau_2^n = \frac{n!}{x_1 k^n} \tag{17}$$

Here $\xi(\in(0,\tau_2))$ $\xi(\in (0,\tau_2))$ is a function of τ_2. To determine the optimal value of ξ, we compared the time delays obtained by using a number of values $[\xi = (0,0.1,0.2,...,1)\tau_2]$ $[\xi = (0, 0.1, \ldots, 1)\tau_2]$ in (17) with those obtained from stochastic simulations. We found that,

when $\xi = 0.5\tau_2$ $\xi = \tau_2/2$, the formula (17) provides more accurate estimate for time delay than other values. In this case, Eq (17) is

$$e^{-k\tau_2/2}\tau_2^n = \frac{n!}{x_1 k^n} \tag{18}$$

The solution of time τ_2 in the above equation is derived from

$$\begin{aligned} \sqrt[n]{e^{-k\tau_2/2}\tau_2^n} &= \sqrt[n]{\frac{n!}{x_1 k^n}} \\ e^{-k\tau_2/(2n)}\tau_2 &= \sqrt[n]{\frac{n!}{x_1 k^n}} \\ e^{-k\tau_2/(2n)}\left(-k\tau_2/(2n)\right) &= \left(-k/(2n)\right)\sqrt[n]{\frac{n!}{x_1 k^n}} \end{aligned} \tag{19}$$

Using the Lambert W function $W(x)$ (y=W(x) if $xe^x = y$), the solution is represented by

$$\begin{aligned} -\frac{k}{2n}\tau_2 &= W\left[-\frac{k}{2n}\left(\frac{n!}{x_1 k^n}\right)^{\frac{1}{n}}\right] \\ \tau_2 &= -\frac{2n}{k}W\left[-\frac{1}{2n}\left(\frac{n!}{x_1}\right)^{\frac{1}{n}}\right] \end{aligned} \tag{20}$$

Now we returned to the general case when $y > 0$. First we consider a particular case with $C_1 = 0$. Then the left hand side of equation (14) is zero. Then we have

$$\tau_2 = \frac{1 + x_1 n - x_1}{k x_1}. \tag{21}$$

Otherwise, the right-hand side of equation (17) is always positive. Using the optimal value $\xi = \frac{t}{2}$, we have that, from Eq. (16),

$$\begin{aligned} \sqrt[n]{e^{-k\tau_2/2}\left(k\tau_2\right)^n} &= \sqrt[n]{C} \\ e^{-k\tau_2/(2n)}\left(k\tau_2\right) &= \sqrt[n]{C} \\ e^{-k\tau_2/(2n)}\frac{-k\tau_2}{2n} &= \frac{-1}{2n}\sqrt[n]{C} \end{aligned} \tag{22}$$

Using the Lambert W function, we have that

$$\begin{aligned} e^{-k\tau_2/(2n)}\frac{-k\tau_2}{2n} &= -\frac{1}{2n}\sqrt[n]{C} \\ \frac{-k\tau_2}{2n} &= W\left(\frac{-1}{2n}\sqrt[n]{C}\right) \end{aligned} \tag{23}$$

and

$$\tau_2 = -\frac{2n}{k}W\left[-\frac{1}{2n}C^{\frac{1}{n}}\right]. \tag{24}$$

In summary, we have an expression for the time delay (11), where the value τ_1 is determined by stochastic simulation algorithm, and

$$\tau_2 = \begin{cases} \dfrac{1+x_1 n - x_1}{k x_1} & if \quad C_1 = 0 \\ -\dfrac{2n}{k} W\left(-\dfrac{1}{2n} C^{\frac{1}{n}}\right) & if \quad C_1 \neq 0 \end{cases}, \tag{25}$$

Note that C_2 C_2 is dependent on the values of $x_1 x_1$, yy, and time tt, which will be determined in Results section by numerical simulations.

Section 3. SSA for calculating time delay

This section provides two algorithms to calculate values of time delay exactly based on the multi-step reaction system (1). For each reaction

$$R_j: \qquad X_j \overset{k}{\rightarrow} X_{j+1}, \tag{26}$$

a propensity function is defined as $a_j = kX_j$ and a stoichiometric vector v_j is defined for the change of molecular numbers due to the firing of this reaction. The SSA is used to determine time delay based on various system conditions [3]. Supplementary Algorithm 1 is used to calculate the delay time for each molecule in the system (1) without any imaginary species initially.

Supplementary Algorithm 1

Step 1: The initial condition is $x_{10} > 0$ and $x_{k0} = 0$ for $k = 2, \ldots, n$ at $t = 0$. The total initial copy number is $s_0 = x_{10}$.

Step 2: Calculate values of propensity function $a_i = k_i x_i$ $(i = 1, \ldots, n)$ and $a_0 = \sum_{i=1}^{n} a_i$.

Step 3: The waiting time μ of the next reaction is determined by

$$\mu = \frac{1}{a_0} ln \frac{1}{r_1}, \tag{27}$$

where $r_1 \sim U(0,1)$.

Step 4: Generate a sample $r_2 \sim U(0,1)$ to determine which reaction with index j from those multi-step reactions will occur, namely

$$\sum_{k=1}^{j-1} a_k(X) < r_2 a_0 \leq \sum_{k=1}^{j} a_k(X) \tag{28}$$

Step 5: Update the system by the determined reaction in step 4

$$X(t+\mu) = X(t) + v_j, \tag{29}$$

if the reaction is the l-th $(l = 1, \ldots, s_0)$ first reaction $X_1 \rightarrow X_2$, then $\tau_{1l} = t + \mu$. If the reaction is the l-th last reaction $X_n \rightarrow P$, then $\tau_{2l} = t + \mu$.

Step 6: If the total copy number $s > 0$, go to step 2. Otherwise, exit the computing loop from step 2 to step 5.

Step 7: Calculate time delay for each moleucle as $\tau_l = \tau_{2l} - \tau_{1l}$ $(l = 1, \ldots, s_0)$..

The following Supplementary Algorithm 2 is used to calculate the delay of the first delay reaction only based on different system states.

Supplementary Algorithm 2

Step 1: The initial condition is $x_{10} > 0$ and $y_0 > 0$. First determine the values of $(x_{20}, \ldots, x_{n0})$ based on the value of y_0, satisfying $x_{20} \geq x_{30} \geq \cdots x_{n0}$ and $x_{i0} - x_{(i+1)0} \leq 1$.

Step 2: Calculate values of propensity function $a_i = k_i x_i$ and $a_0 = \sum_{i=1}^{n} a_i$.

Step 3: The waiting time μ of the next reaction is determined by (27).

Step 4: Generate a sample $r_2 \sim U(0,1)$ to determine which reaction with index j from those multi-step reactions will occur (28).

Step 5: Update the system by the determined reaction in step 4

$$X(t+\mu) = X(t) + v_j. \tag{30}$$

If it is the first firing of reaction $X_1 \rightarrow X_2$, then $\tau_1 = t + \mu$. If it is the last reaction $X_n \rightarrow P$ to let the total copy number be $x_{10} - 1$, then $\tau_2 = t + \mu$. Then exit computing loop from step 2 to step 5 and calculate time delay $\tau = \tau_2 - \tau_1$.

Note that the values of time delay obtained from Algorithms 1 and 2 are stochastic. The averaged value of time delay from a large number of simulations will be used to match the deterministic values of τ_2 in (25).

References

1. Tian, T.H., *Simplified stochastic models with time delay for studying the degradation process of mRNA molecules.* International Journal of Data Mining and Bioinformatics, 2014. **10**(1): p. 18-32.
2. Leier, A., T.T. Marquez-Lago, and K. Burrage, *Generalized binomial tau-leap method for biochemical kinetics incorporating both delay and intrinsic noise.* The Journal of chemical physics, 2008. **128**(20): p. 205107.
3. Gillespie, D.T., *Exact Stochastic Simulation of Coupled Chemical-Reactions.* Journal of Physical Chemistry, 1977. **81**(25): p. 2340-2361.

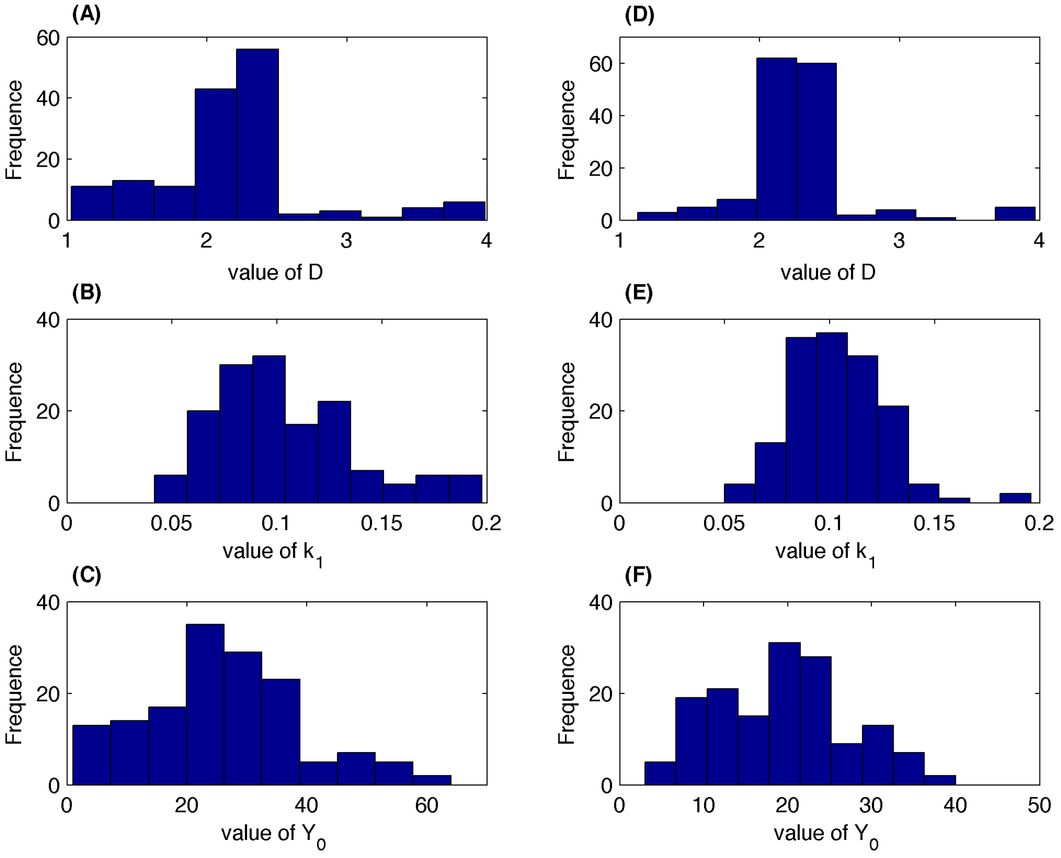

SFigure 1. Distributions of estimated model parameters for gene *RPL30* degradation. (A, B, C) Parameters y_0, *D* and *k* for construct ACT1, respectively. (D, E, F) Parameters y_0, *D* and *k* for construct RPL30, respectively.

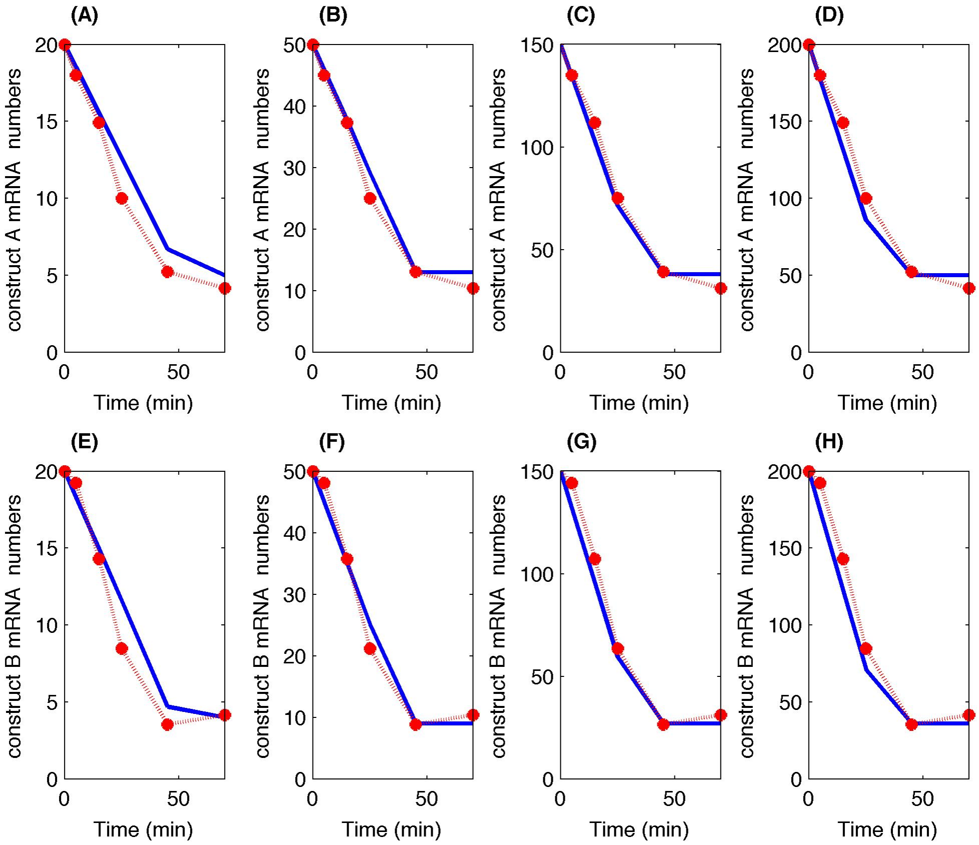

SFigure 2. Simulation of mRNA degradation for gene RPL30 using different initial mRNA number s_0 s_0**.** For each construct, parameters k and D are the same as those in Figure 3. The value of y_0 is proportional to the value of s_0. (A, B, C, D) Construct ACT1. (E, F, G, H) Construct RPL30. (A, E) $s_0 = 20$ $s_0 = 20$. (B, F) $s_0 = 50$ $s_0 = 50$. (C, G) $s_0 = 150$ $s_0 = 150$. (D, H) $s_0 = 200$ $s_0 = 200$. (Solid-line: simulation. Dash-dot line: experimental data).

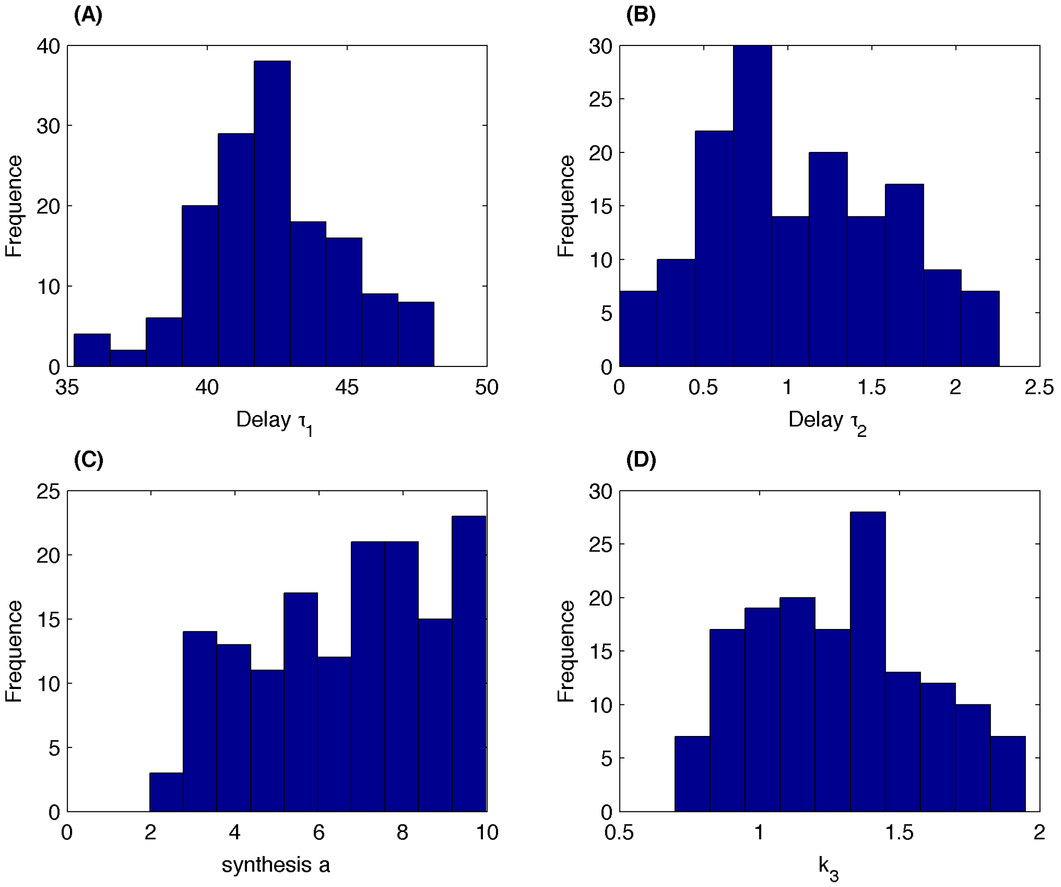

SFigure 3. Distributions of estimated model parameters for gene *SWI5* transcription.
(A) Transcription delay τ_1. (B) Translocation delay τ_2. (C) Synthesis parameter a. (D) Degradation rate constant k_3.

STable 1. Simulation error of the one-step, two-variable and state-dependent delay models. EB: error bar.

	One-step	Two-variable	SD delay
Error for construct (A)	20.54	17.47	13.02
Error for construct (B)	39.12	26.58	19.35
Number beyond EB (A)	3	2	1
Number beyond EB (B)	4	3	1
